# Targeted Anthocyanin Profiling of Fruits from Three Southern Highbush Blueberry Cultivars Propagated in Colombia

**DOI:** 10.3390/molecules29030691

**Published:** 2024-02-02

**Authors:** Jessica Prada-Muñoz, Ericsson Coy-Barrera

**Affiliations:** Bioorganic Chemistry Laboratory, Universidad Militar Nueva Granada, Cajicá 250247, Colombia; jessipra1217@gmail.com

**Keywords:** blueberry, phenolics, flavonoid, anthocyanin, antioxidant, targeted analysis

## Abstract

The blueberry, a deciduous shrub in the Ericaceae family, is celebrated for its delightful flavor, sweetness, and abundance of anthocyanins and antioxidants, qualities that have garnered significant attention for their potential health benefits. Blueberries grown in diverse environments and exhibit varied anthocyanin profiles, often influenced by factors such as altitude and climate. Varietal groups worldwide have been bred and categorized based on their growth habits and specific cold requirements, particularly with southern highbush cultivars thriving in temperate climates, demonstrating tolerance to higher altitudes or cooler climates—a result of hybridizations involving various *Vaccinium* species. In the Colombian Andes, southern highbush blueberries thrive in unique high-altitude conditions, leading to exceptional quality due to the region’s cool climate and specific soil characteristics. In this context, this study aimed to chemically characterize and differentiate three southern highbush blueberry cultivars (i.e., ‘Biloxi,’ ‘Legacy’ and ‘Sharpblue’) cultivated in a Colombian Andean plateau and compare them to three commercially available highbush blueberries. This comprehensive evaluation involved examining total phenols, flavonoids, anthocyanin content, and DPPH· free-radical scavenging capacity, as well as conducting anthocyanin-targeted profiling via HPLC-DAD-HRMS. Through supervised multivariate analyses such as sPLS-DA, this study delved into the pattern recognition of those anthocyanins that could potentially serve as markers for quality and cultivar-related chemical trait determination. These findings locate blueberry-derived anthocyanins in a metabolic context and afford some insights into southern highbush blueberry cultivar differentiation to be used for further purposes.

## 1. Introduction

Edible fruits are abundant in phenolics and anthocyanins, which are invaluable contributors to a balanced and healthful lifestyle [1]. These compounds, found in fruits like blueberries, blackberries, cherries, and raspberries, serve as potent antioxidants, actively combating oxidative stress and inflammation within the body [2,3]. They enhance immune function and cardiovascular health and exhibit the potential to reduce the risk of chronic diseases such as cancer and neurodegenerative conditions [4,5]. Incorporating these nutrient-dense fruits into one’s diet adds vibrant flavors and harnesses their nutraceutical properties for overall well-being and a bolstered defense against various health challenges [6].

Highbush blueberries stand out among the most renowned fruits recognized for their notably high anthocyanin content. The Ericaceae family houses the blueberry within the *Vaccinium* genus, comprising around 450 species found worldwide, primarily concentrated in the Northern Hemisphere [7]. A known representative is *Vaccinium corymbosum*, which typically forms a crown and grows up to 3 m in height, characterized by two to five stems originating from a single hole and possessing deep-fibrous roots that don’t penetrate dense soils, rendering it sensitive to drought [8,9]. Its fruits, usually juicy, sweet, and spherical in a blue-black hue, measure around 7 to 10 mm in diameter and contain small seeds [10,11]. Covered by a specific waxy epidermis, this plant underwent domestication in 1906, with a rapid development period when grown in field conditions (ca. 90 days), leading to improved fruit quality [9,12]. 

Often classified as a functional food, highbush blueberries possess health-promoting attributes [13,14,15,16]. Its consumption has been associated with the reduction of various diseases such as cancer, cardiovascular diseases, Alzheimer’s, Parkinson’s, hypertensive brain damage, muscular dystrophy, multiple sclerosis, anemia, hepatitis, and aging [17]. This positive impact is attributed to its high content of anthocyanins, flavonoids, and polyphenols, which exhibit antioxidant properties (ca. 3-fold higher than strawberries or raspberries), mitigating the adverse effects of oxidative stress induced by free radicals [18,19,20]. In this regard, anthocyanins have garnered significant interest due to their natural dye properties, which are responsible for the vibrant red-purple hues in various fruits and flowers [21]. Typically found in the vacuole solution within epidermal and subepidermal cells or specialized regions known as anthocyanoplasts primarily in the epicarp and to a lesser extent in the mesocarp of fruits [22], blueberries boast around fifteen distinct anthocyanins, primarily derived from the well-known cyanidin, peonidin, delphinidin, petunidin, and malvidin cores [13,23]. These compounds exhibit high stability at low pH levels but require protection from sunlight to preserve their antioxidant properties [24].

The anthocyanin content in blueberries exhibits intriguing variations contingent upon their cultivation in diverse environmental settings, particularly under domestication [25,26]. The distinct geographic locations, encompassing varying temperatures, sunlight exposure, soil compositions, and altitude, significantly influence the synthesis and concentration of anthocyanins in blueberries [27]. Studies have revealed that blueberries grown in different environments tend to display nuanced anthocyanin profiles, with fluctuations in the abundance of specific anthocyanin types such as cyanidin, delphinidin, peonidin, and malvidin [28]. Higher altitudes or cooler climates often foster increased anthocyanin production, contributing to deeper hues and potentially elevated antioxidant properties [27]. As a result, blueberries introduced for cultivation in the Colombian Andes flourish in the unique high-altitude environments of the region. The cool climate and specific soil conditions in the Andean foothills provide an ideal setting for these berries, contributing to their exceptional quality [29,30]. The Andean cultivation of exotic blueberries represents an opportunity for local economies, providing sustainable agricultural practices and fostering economic growth within these communities [30]. Furthermore, this introduction offers context for the exploitation of native wild species, commonly known as Andean blueberries, predominantly comprising *V. meridionale* or *V. floribundum* [31]. They are recognized for yielding smaller-sized fruits with a subtly acidic flavor, contributing a delightful nuance to various preparations when compared to highbush blueberries.

In this context, breeding programs have been conducted to adapt blueberries to diverse regional conditions while enhancing key traits such as vigor, disease resistance, fruit yield, flavor, firmness, and chilling requirements [32]. Consequently, varietal groups globally have been selectively bred and classified according to their growth habits and distinct cold requirements. In this categorization, the southern highbush blueberry cultivars thrive in temperate climates, tolerating higher temperatures while requiring 200 to 600 cold hours [33,34]. They are hybrids resulting from crossings primarily involving *V. corymbosum*, *V. elliottii*, *V. ashei*, and *V. darrowi* [35,36,37]. These cultivars are suited for latitudes between 28 and 35 degrees, where low temperatures seldom drop below 7 °C. They find their primary cultivation areas in Florida, South Georgia, northern Chile, southern Spain, Colombian Andes, and presently, in northern Africa. Known for their early behavior, most hybrids boast an extended harvest period, initiating flowering and budding quite early, and consequently, they face a significant risk of frost damage [38]. Notable cultivars among these hybrids include ‘O’Neal’, ‘Biloxi’, ‘Emerald’, ‘Jewel’, ‘Legacy’, ‘Misty’, ‘Sharpblue’, and ‘Star’. In Colombia, ‘Biloxi’ and ‘Sharpblue’ stand as the most encouraged cultivars, characterized by upright, vigorous growth patterns and high productivity [39]. 

The introduction of southern highbush blueberry cultivation in the Colombian Andean has provided valuable insights into crop yield and fruit quality traits of blueberry cultivars [30]. However, there remains a dearth of data concerning the composition of phenolics and anthocyanins, along with potential variations attributable to the specific growing conditions of the Andean plateaus. This study aims to delineate differences in phenolic content and anthocyanin profiles among fruits from three locally cultivated blueberry cultivars (‘Biloxi’, ‘Legacy’, and ‘Sharpblue’). This research constitutes the comparative chemical characterization of these cultivars, contrasted with commercially available fruits, marking it the first such evaluation in this context.

## 2. Results and Discussion

### 2.1. Total of Phenolics, Flavonoids, and Anthocyanins and Antioxidant Capacity

Phenolics in edible berries represent a powerhouse of health-promoting compounds [40]. These phenolic compounds, including flavonoids and anthocyanins, not only contribute to the vibrant colors and distinctive flavors of berries but also possess potent antioxidant properties [40,41]. Beyond their sensory contributions, these compounds offer various health benefits, such as reducing oxidative stress, combating inflammation, and potentially mitigating chronic disease risks. The rich phenolic content in berries underscores their nutritional value, making them valuable additions to a balanced diet for overall health and wellness [42]. Consequently, despite the recognized limitations, plausible interferents, and drawbacks, the so-called total quantification of these compounds via spectrophotometric analysis constitutes an initial approach since it provides relevant comparative insights into the overall metabolite content through relatively straightforward procedures [43,44].

**Figure 1:**
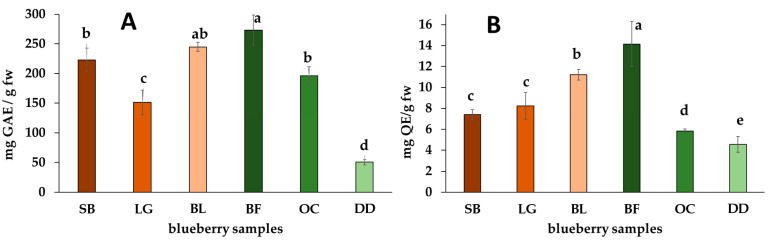
Total phenolic content (TPC) (**A**) and total flavonoid content (TFC) (**B**) for commercially available and locally cultivated fruit samples of blueberries. Commercial berries = Berry Fruit (BF), Delynat Dried (DD), and Ocati (OC); Locally cultivated southern highbush blueberry cultivars = ‘Legacy’ (LG), ‘Sharpblue’ (SB), and ‘Biloxi’ (BL). Data expressed as means ± standard deviation (*n* = 6). Different lowercase letters over bars indicate significant differences according to the Tukey test. TPC and TFC expressed per gram of fresh basis (fw).

Under this context, the total phenolic content (TPC), total flavonoid content (TFC), and total anthocyanin content (TAC) were then quantified to discern those differences from the gathered blueberry samples. Hence, the content variations between fruits locally sourced from Guasca-Cundinamarca, Colombia, such as fruits from ‘Legacy’ (LG), ‘Sharpblue’ (SB) and ‘Biloxi’ (BL) cultivars) and commercially purchased, imported blueberries (i.e., fresh Berry Fruit (BF), Ocati (OC), and dehydrated Delynat (DD) fruits) allowed discrimination based on such chemical traits. Accordingly, the commercial samples exhibited the most considerable variability in TPC and TFC (Figure 1). 

Notably, the dehydrated blueberry samples (DD) showed the lowest phenol and flavonoid levels (i.e., 57.8 ± 23.2 and 4.57 ± 0.78 mg QE/g fw, respectively), while the commercially available BF blueberry exhibited significantly higher levels in this regard (i.e., 273.1 ± 25.4 and 14.2 ± 2.1 mg QE/g fw, respectively). Conversely, locally cultivated samples demonstrated comparatively lesser fluctuations in TPC and TFC levels. However, the BL cultivar exhibited the highest TPC and TFC values among locally cultivated fresh berries, while LG and SB showed lower phenolic and flavonoid contents, respectively.

**Figure 2:**
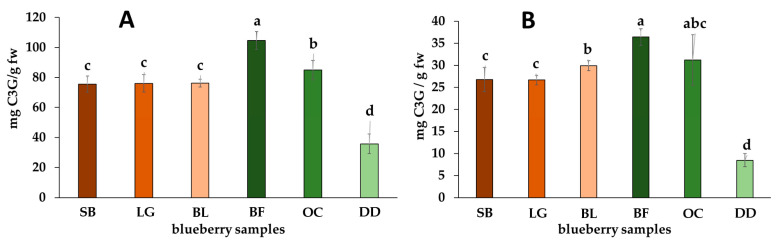
Total anthocyanin content (TAC) evaluated by (**A**) pH-differential and (**B**) AlCl_3_-complexing methods for commercially available and locally cultivated fruit samples of southern highbush blueberry cultivars. Commercial berries = Berry Fruit (BF), Delynat Dried (DD), and Ocati (OC); Locally produced cultivars = ‘Legacy’ (LG), ‘Sharpblue’ (SB), and ‘Biloxi’ (BL). Data expressed as means ± standard deviation (*n* = 6). Different lowercase letters over bars indicate significant differences according to the Tukey test. TAC expressed per gram of fresh basis (fw).

The process of drying for obtaining dehydrated fruits (e.g., DD) seems to contribute to a reduction in phenolic compound levels, potentially stemming from exposure to elevated temperatures, consequently diminishing the phenolic-related fruit’s quality compared to its fresh counterparts [45]. Many of these compounds, accountable for color, aroma, taste, and the preservation of fats, vitamins, and enzymes, might undergo degradation under high-temperature conditions [46,47]. Conversely, fresh commercial samples, such as BF, demonstrated a higher level of metabolite preservation, possibly due to continuous cold postharvest storage practices that maintain their inherent properties intact [48,49]. However, a previous study on *V. corymbosum* berries (‘Ventura’ cultivar) evaluated dehydration via air drying, revealing an increase in phenolic compound content during the drying process. The highest content was observed at the highest temperature, showing optimal results at 50 °C [50]. Therefore, the impact of dehydration on phenolic and anthocyanin contents depends on the drying method. A comparison of different dehydration techniques (e.g., osmotic dehydration, freeze-drying, convection drying) revealed the least impact with convection drying [51]. The dehydrated DD sample also demonstrates the lowest TAC across both methods (Figure 2), hinting at possible anthocyanin degradation during the drying process, which remained unknown for this commercial sample [52]. Within fruits, anthocyanins find stability through metallic ions and organic compounds like citric and ascorbic acid. However, exposure to higher temperatures and light may lead to their degradation and impact their integrity [53].

The measured TPC values in the established Colombian blueberry cultivars were lower in comparison to studies conducted on northern and southern highbush blueberry cultivars (*n* = 15) from three locations in the USA, where TPC values ranged from 300 to 700 mg GAE/100 g fw [54]. Interestingly, three blueberry cultivars produced in South Korea exhibited TPC values similar to our findings (ranging from 174 to 283 mg GAE/100 g fw), contingent upon the production system (i.e., heated, open field, non-heated) [55]. Additionally, five southern highbush blueberry cultivars established in Georgia demonstrated values ranging from 261 to 585 mg GAE/100 g fw [56]. In this context, the ‘Legacy’ (LG) cultivar in the USA exhibited lower TPC values than that of our study, ranging from 414 to 518 mg GAE/100 g fw [54]. Notably, the ‘Sharpblue’ cultivar displayed lower values compared to blueberries from Georgia (i.e., 583 mg GAE/100 g fw) [56] but similar to those reported in South Korea (246–270 mg GAE/100 g fw) [55]. Moreover, TPC values measured in berries from the ‘Biloxi’ (BL) cultivar established in Mexico showed a range of 307 to 597 mg GAE/100 g fw, dependent on the harvest season [57]. These variations across different cultivation regions worldwide suggest influences from diverse factors such as location-specific environmental conditions, seasonal variations, climate nuances, and even the production system [54,55,56,57], which can also impact the antioxidant properties of the blueberries. Understanding these differences could offer insights into optimizing cultivation practices and environmental conditions to potentially enhance the phytochemical content in blueberry cultivars produced in Colombia.

On the other hand, anthocyanins were quantified using two methods, i.e., the pH-differential and the AlCl_3_-complexing methods. The latter particularly targets *o*-hydroxylated or C5-hydroxylated anthocyanins, allowing for nuanced observations between samples [58]. The outcomes of these analyses are illustrated in Figure 2. The commercially available BF berries also exhibit higher TAC (0.10 ± 0.01 mg C3G/g and 36.41 ± 1.91 mg C3G/g) using both methods, while the dried sample DD reports the lowest values (0.08 ± 0.01 mg C3G/g and 8.49 ± 1.51 mg C3G/g). These differences underscore the impact of processing methods and storage conditions on the retention of anthocyanins, which play a pivotal role in the perceived quality and health benefits of blueberries. Further exploration of these findings could elucidate optimal storage and processing practices to preserve the bioactive compounds crucial for the fruit’s nutritional value and consumer appeal. Remarkably, locally cultivated blueberries exhibited similar TAC values between them by the two methods but lower than those reported in other countries (TAC > 60 mg C3G/g fw) via the pH-differential method [54,55,56,57].

**Figure 3:**
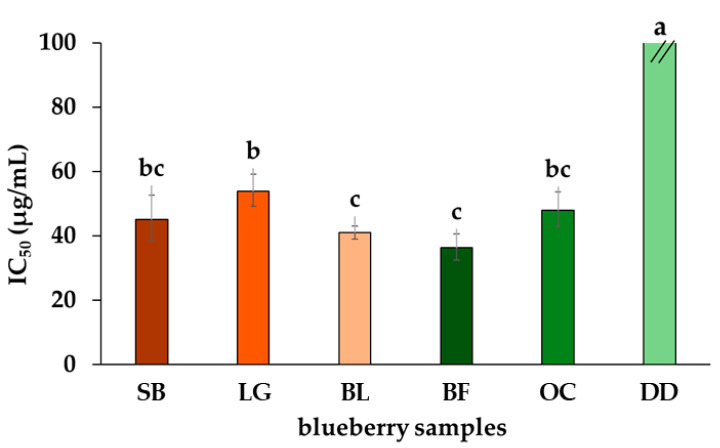
DPPH free radical scavenging assay for commercially available and locally cultivated fruit samples of southern highbush blueberry cultivars. Commercial berries = Berry Fruit (BF), Delynat Dried (DD), and Ocati (OC); Locally cultivated southern highbush blueberry cultivars = ‘Legacy’ (LG), ‘Sharpblue’ (SB), and ‘Biloxi’ (BL). Data expressed as means ± standard deviation (*n* = 6). Different lowercase letters over bars indicate significant differences according to the Tukey test.

Finally, the well-documented antioxidant capacity of phenols, flavonoids, and anthocyanins positions blueberries as an enticing source of these beneficial compounds, elevating their status as a functional food [59]. The DPPH free radical scavenging method was employed to evaluate the antioxidant capacity of the test blueberries (Figure 3). The radical scavenging was conducted on acidified methanolic extracts, as anthocyanins exhibit increased stability under acidic pH conditions, ensuring their integrity for subsequent antioxidative evaluation. Additionally, the DPPH method at acidic pH, despite potential pH-related deviations, fosters the dominance of the proton-coupled electron transfer (PC-ET) mechanism [60], preventing mixed antioxidant actions by the anthocyanin-rich extract.

As depicted, dehydrated fruits exhibit the poorest outcome again, i.e., the highest measured half-maximal inhibitory concentration (IC_50_ = 478 µg/mL). This value stands out starkly, being nearly ten times higher than the average IC_50_ of the other samples. Surprisingly, this huge difference aligns with the significant distinctions observed among the samples examined through the total contents of antioxidant compounds. The other commercial samples (OC and BF) exhibited similar IC_50_ values to locally produced cultivars (36 µg/mL < IC_50_ < 54 µg/mL). This suggests a uniformity in this specific aspect of their antioxidant properties, which could be an intriguing area for deeper investigation or comparative studies in the future. Regarding the SB, LG, and BL cultivars, the better antioxidant capacity was observed in the BL cultivar (IC_50_ = 41 µg/mL), whereas the LG cultivar exhibited the highest IC_50_ value (=53 µg/mL). These findings highlight the potential impact of drying methods on the antioxidant capacity of blueberries, indicating potential directions for further research to refine preservation techniques and maintain the valuable antioxidants.

### 2.2. Targeted Anthocyanin Targeted Anthocyanin Profiling-Based Differentiation

**Figure 4:**
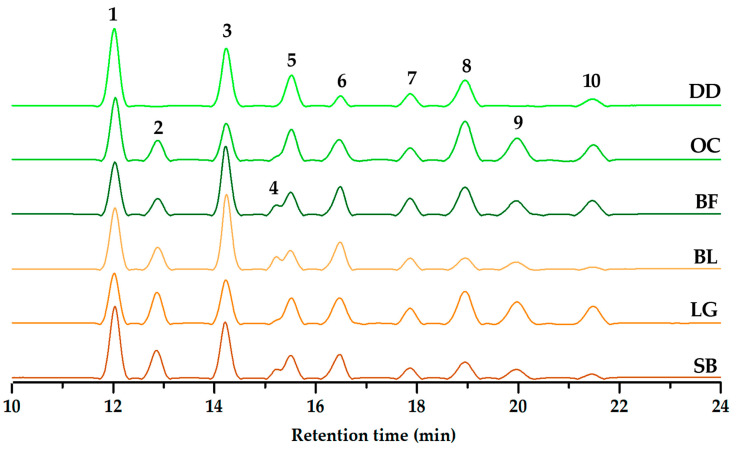
Stacked chromatographic profiles of extracts from commercially available and locally cultivated blueberry fruit samples (monitored at 520 nm). Commercial berries = Berry Fruit (BF), Delynat Dried (DD), and Ocati (OC); Locally cultivated southern highbush blueberry cultivars = ‘Legacy’ (LG), ‘Sharpblue’ (SB), and ‘Biloxi’ (BL). Numbers over chromatographic peaks are related to the detected compounds in the HPLC analysis.

The potential impact of unconsidered metabolites on antioxidant capacity prompted LC-HRMS analysis, aiming for a comprehensive understanding of sample composition, complexity, and metabolite identification. In this regard, Figure 4 showcases the chromatographic profile (HPLC-DAD-ESI-HRMS) of the blueberry-derived extracts. Hence, fresh samples revealed ten peaks associated with anthocyanins, while DD exhibited a notably reduced number of peaks (*n* = 7). This observation aligns with the deduced notion of plausible compound degradation during dehydration, indicating a potential influence on the anthocyanin profile. 

For further clarity, Table 1 offers a concise summary detailing the chromatographic profile, retention times, and the annotation of each identified peak. Unlike other fruits, blueberries possess a distinct characteristic with only four common anthocyanin aglycone cores: cyanidin, delphinidin, malvidin, and petunidin. Additionally, the diagnostic analysis based on mass spectrometry (MS) combined with chromatographic behavior suggested the presence of two hexoses (glucose and galactose) and one pentose (arabinose) as sugar residues in the detected anthocyanins [55]. These specific anthocyanins have been consistently reported in both the investigated southern highbush blueberry cultivars and others in the literature [26,55,61]. However, a lower count of detected anthocyanins (*n* = 10) was identified in the Colombian-produced cultivars compared to previous reports, where the number ranged from 11 to 18 detected compounds [26,55]. Conversely, the SB cultivar in South Korea showed a similar count (*n* = 11), differing in the presence of delphinidin arabinoside, which was not detected in our study for the SB cultivar. This divergence in anthocyanin composition emphasizes the unique profile of blueberries, potentially influencing their distinct health-promoting properties and color variations. Interestingly, while the three locally produced cultivars exhibited the same number of anthocyanins, they differed in relative abundance. Delving deeper into these metabolites could pave the way for improved preservation techniques, ensuring the retention of bioactive compounds vital for blueberry quality and nutritional benefits. Further exploration using LC-HRMS analysis holds promise in uncovering additional compounds and understanding their roles in the antioxidant properties of southern highbush blueberries.

The quantitative analysis delved into determining the individual content of each anthocyanin, as detailed in Table 2, measured in milligrams equivalent to cyanidin-3-glucoside per 100 g of fresh weight (mg C3G/100 g fw) using an external standard. The results revealed the highest abundance in delphinidin galactoside (**1**) and cyanidin glucoside (**3**), with contents ranging from 79.8 to 115 mg C3G/100 g fw and 59.7 to 110.5 mg C3G/100 g fw, respectively. Following closely was malvidin galactoside (**8**), observed within a range of 25.4 to 88.2 mg C3G/100 g fw. Conversely, cyanidin glucoside (**4**), petunidin arabinoside (**7**), and malvidin (**10**) arabinoside exhibited lower abundance, ranging from 4.3 to 13.5 mg C3G/100 g fw, 16.9 to 30.3 mg C3G/100 g fw, and 7.6 to 42.1 mg C3G/100 g fw, respectively. Notably, the dehydrated berry DD displayed distinct accumulations of particular anthocyanins (e.g., **1** and **5**) with significantly different contents compared to the other test blueberries. On the contrary, the LG and BL cultivars showcased higher contents for specific anthocyanins, such as **2**, **6**, **7**, **9**, and **10** for LG and **3** and **4** for BL.

**Figure 5:**
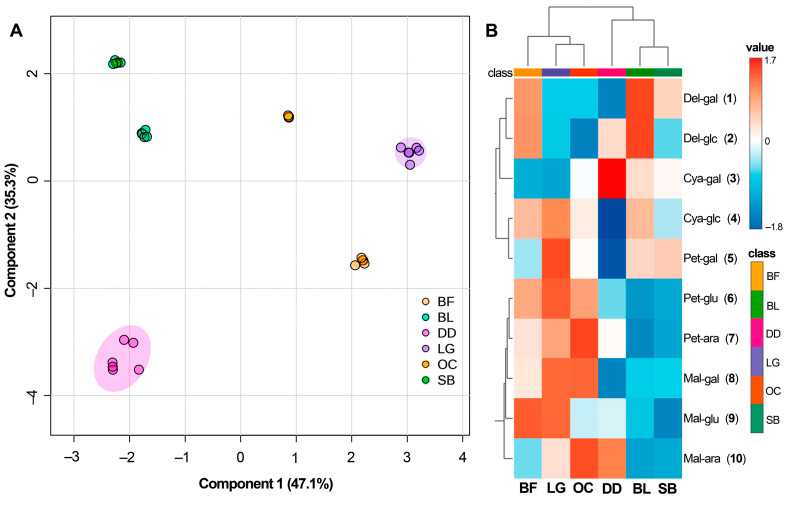
(**A**) PC1 vs. PC2 score plot derived from the partial least squares-discriminant analysis (PLS-DA) on the quantitative dataset of anthocyanins quantified from three southern highbush blueberry cultivars. The sPLS-DA model was built using the quantitative data from the commercial and cultivated blueberries. Commercial berries = Berry Fruit (BF), Delynat Dried (DD), and Ocati (OC); Locally cultivated southern highbush blueberry cultivars = ‘Legacy’ (LG), ‘Sharpblue’ (SB), and ‘Biloxi’ (BL). 95% confidence represented by purple and pink ellipsoids. (**B**) Distribution of the quantitative data of the test anthocyanins in extracts of blueberries. The heat map is organized by columns for each blueberry. Each color cell is associated with a normalized (scaled to unit variance, prior heatmap generation) content (mg cyanidin-3-glucoside/100 g fresh weight) of each anthocyanin, depending on the color scale (dark red = positive correlation; dark blue = negative correlation). The data are organized according to the Ward clustering algorithm measuring Euclidean distance, and numbered according to the annotated metabolite list (Table 1).

To unravel the connections among the recorded anthocyanin profiles, a sparse partial-least square-discriminant analysis (sPLS-DA) was conducted using the quantitative data presented in Table 2. The model effectively accounted for 82.4% of the data variance, indicating a robust explanatory analysis. The resulting scores plot (Figure 5A) illustrated that BL and SB cultivars share similar anthocyanin profiles, setting them apart from the LG cultivar, which showed a closer association with the commercial blueberries BF and OC. 

Notably, the DD samples formed a distinct cluster in a separate quadrant, indicating more pronounced data dispersion. These clusterings indicated subtle yet discernible differences in chemical composition among studied blueberries, which are noteworthy although not statistically significant for practical applications [63]. This distinction was better visualized in the heat map (Figure 5B), offering an intuitive display of each anthocyanin’s content. The heat map, generated through ward-mediated clustering, revealed a grouping where BF, OC, and SB clustered together due to their higher accumulation of compounds **6**–**9**. Conversely, the other cluster comprised DD, BL, and SB, primarily characterized by the prevalence of anthocyanins **1**–**4**.

**Figure 6:**
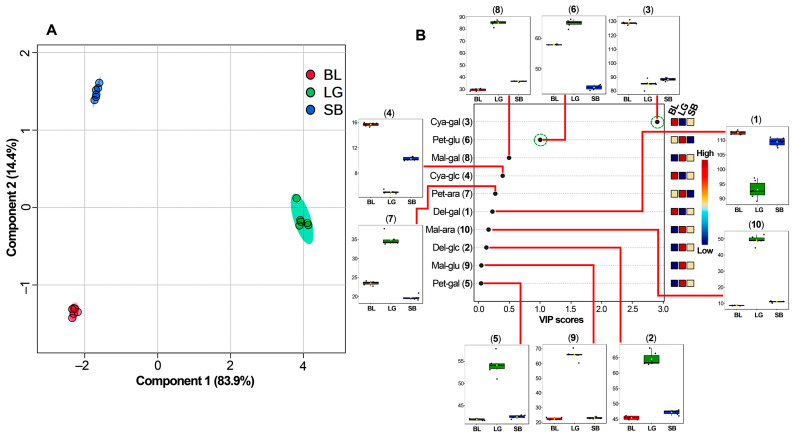
(**A**) PC1 vs. PC2 score plot and (**B**) Variable importance in projection (VIP) plot derived from the partial least squares-discriminant analysis (PLS-DA) on the quantitative dataset of anthocyanins quantified from three southern highbush blueberry cultivars. The sPLS-DA model was built using the quantitative data from the three cultivars. Cultivars = ‘Legacy’ (LG), ‘Sharpblue’ (SB), and ‘Biloxi’ (BL). Numbering and annotation of quantified anthocyanins according to Table 1. Anthocyanin contents expressed as mg eq a cyanidin-3-glucoside (C3G) per 100 g fresh weight (fw). Each colored square on the VIP plot’s left side indicates the highest (dark red) or lowest (dark green) relation between the seed sample and compound. The highly top-ranked anthocyanins are highlighted in green dashed circles. Each anthocyanin is connected through a red line to the respective box plot to represent the data distribution among cultivars.

A supplementary sPLS-DA exploration was also conducted to discern the distinctive differences among the three locally cultivated southern highbush blueberry cultivars. The model effectively explained 98.1% of the data variance, indicating its robustness for predicting. The resulting scores plot (Figure 6A) revealed three distinct groups corresponding to each cultivar replicate (*n* = 6). Principal Component 1 (PC1) showcased a significant separation between the LG cultivar and the BL and SB cultivars (83.9% variance), indicating a pronounced difference. Meanwhile, Principal Component 2 (PC2) differentiated the BL and SB cultivars (14.4% variance).

A more in-depth analysis for pattern recognition was facilitated through the sPLS-DA-derived variable importance in the projection (VIP) plot (Figure 6B). This provided a nuanced understanding of how specific variables influenced sample clustering across both principal components (PC1 and PC2). The VIP scores delineated the anthocyanin contents responsible for chemically mediated cultivar discrimination. Notably, two anthocyanins exhibited VIP scores > 1.0, signifying their substantial relevance in categorizing a particular cultivar in the Colombian Andean plateau. Specifically, cyanidin galactoside (**3**) was identified as a discriminant for the BL cultivar (VIP ca. 3.0), while petunidin glucoside (**6**) was the most differential compound for LG cultivar. Consequently, these specific variables emerge as promising markers, holding potential significance in tracking and determining the origin and chemotype of the fruit [63,64]. Interestingly, the SB cultivar did not exhibit an anthocyanin specifically accumulated in fruit extracts for discriminatory purposes between the investigated cultivars. Other anthocyanins exhibited patterns related to the BL or LG cultivar, with a VIP below 1.0, indicating low relevance for discrimination.

These anthocyanin-dependent patterns contribute to understanding the intricate interplay of compounds and facilitate sample differentiation based on their chemical profiles [65]. Supervised methods like sPLS-DA, based on fundamental chemical features, emerge as valuable diagnostic tools for assessing fruit quality and origins, thus holding considerable significance in commercial contexts [63,66]. Therefore, if the production and variability of anthocyanins demonstrate dependency, the presence of specific anthocyanin accumulation could potentially serve as indicative markers [67,68]. This observation hints at the prospect of using specific anthocyanin profiles as potential indicators to infer the geographical or varietal origins of blueberry samples—a facet crucial in authentication and quality assessment protocols [64]. Further investigations correlating specific anthocyanins with geographical regions, production systems, or cultivars could significantly enhance the precision and reliability of authenticity testing for blueberry products.

Additionally, the distinct clustering based on these variables signifies their robust discriminatory power, showcasing their capacity to differentiate between local and imported samples. This identification of discriminative markers offers valuable insights into potential tools for authentication and quality assessment protocols. Further exploration and validation of these markers across a broader sample set could fortify their applicability in confirming the origin and varietal identity of blueberry samples, thereby bolstering the reliability and precision of quality assessment methodologies.

## 3. Materials and Methods

### 3.1. Plant Material

Fruits from locally cultivated plants in the Guasca district (Cundinamarca, Colombia, coordinates: 4.854248, −73.883731) were obtained from three southern highbush blueberry cultivars (‘Legacy’ (LG), ‘Sharpblue’ (SB), and ‘Biloxi’ (BL)). The location has a subtropical highland climate (Cfb) per the Köpen-Geiger classification, with fruits randomly collected from 24-month-old plants in a commercial open crop (temperature = 13 ± 3 °C, relative humidity = 78 ± 6%, altitude = 2562 m.a.s.l., mean annual precipitation = 640.8 mm with a bimodal behavior). Additionally, blueberries were purchased from local markets, including imported fruits such as Berry Fruit (BF), Delynat Dehydrated (DD), and Ocati (OC).

### 3.2. Sample Preparation

Fruits (500 mg) were finely ground in liquid nitrogen, macerated, and extracted in acidic methanol (1% HCl) overnight with continuous stirring in a orbital shaker (90 rpm). All procedures were conducted under low light conditions. The resulting solution was filtered and adjusted to a final volume of 25.0 mL using a volumetric flask. Each extraction process was performed in triplicate for outcome consistency and reliability.

### 3.3. Total Phenolic Content (TPC)

The TPC of the prepared extracts was determined using the Folin–Ciocalteu method [69]. Initially, 200 µL of the extract underwent dilution to 600 µL with distilled water and was combined with 400 µL of Folin-Ciocalteu’s reagent. After a 3-min incubation, 1500 µL of a 7.35% (*w*/*v*) aqueous Na_2_CO_3_ solution was added. The reaction occurred for two hours in the absence of light, and the absorbance was measured at 765 nm. TPC was quantified using gallic acid equivalents (GAE) derived from a calibration curve, and the results were expressed as milligrams of GAE per gram of fresh basis (mg GAE/g fw).

### 3.4. Total Flavonoid Content (TFC)

The TFC of the prepared extracts was evaluated utilizing the AlCl_3_ complexing method with modifications [70]. In brief, an aliquot of the extract (1000 μL) was combined with ethanol (800 μL), 10% aluminum chloride (200 μL), and 0.1 M sodium acetate (200 μL). The mixture was vortexed and kept in darkness for 40 min, and the absorbance was measured at 420 nm. Each measurement was performed in triplicate. TFC was quantified using quercetin equivalents (QE) derived from a calibration curve, and the results were expressed as milligrams of QE per gram of fresh basis (mg QE/g fw).

### 3.5. Total Anthocyanin Content (TAC)

The TAC of the prepared extracts was evaluated through the pH-differential method [71]. Briefly, an aliquot (1500 μL) of each extract was mixed separately with pH 1 buffer (1500 μL, 0.025 M potassium chloride), and another aliquot (1500 μL) was added separately to pH 4.5 buffer (1500 μL, 0.4 M sodium acetate). Absorbance was measured at 520 and 700 nm, respectively. Each measurement was performed in triplicate. TAC was quantified using cyanidin-3-*O*-glucoside (C3G) equivalents, and the results were expressed as milligrams of C3G per gram of fresh basis (mg C3G/g fw) using Equation (1) as follows:

(1) $$\mathrm{mg}\;C3G/g\;\mathrm{fw}=\frac{A\;\times \;\mathrm{MW}\;\times \;\mathrm{DF}\;\times \;V\;\times \;10^{3}}{\varepsilon \;\times \;l\;\times \;\mathrm{SW}}$$

where A = (A_520nm_ − A_700nm_)_pH 1.0_ − (A_520nm_ − A_700nm_)_pH 4.5_; MW (molecular weight) = 449.2 g/mol for C3G; DF = dilution factor; V = total volume of sample solution after extraction, in L; 10^3^ = factor for conversion from g to mg; ε (molar extinction coefficient) = 26,900 L·mol^−1^·cm^−1^, for C3G; *l* = path length, in cm; and SW = sample weight used for extraction, based on 100 g dried material. In addition, the anthocyanin content was also determined by the Al complexing method [58], following the same procedure for TFC, except for the absorbance measurement at 520 nm and employing cyanidin-3-*O*-glucoside as standards. The Al-complexed anthocyanin content was also expressed as mg C3G/g fw.

### 3.6. DPPH· (1,1-Diphenyl-2-picryl-hydrazyl) Radical Scavenging Assay

The antioxidant activity was assessed using the DPPH· radical scavenging assay, as described by a previously reported method [72]. A working solution was prepared by diluting a 100 mM stock of DPPH in methanol to achieve an absorbance close to 1 at 515 nm. Subsequently, increasing volumes of AR extract solutions (1–15 µL) were added to each well of a 96-well microplate, with 96% ethanol adjusted to complete 25 µL per well. Following this, DPPH· working solution (175 µL) was added to each well, and the plate was incubated in darkness for 1 h at room temperature. Each extract concentration was measured in triplicate alongside a control consisting of DPPH· (175 µL) and ethanol (25 µL). Absorbance readings at 515 nm were then recorded. The radical percentage of DPPH· scavenging was determined using the following formula: %DPPH scavenging = [(control absorbance − sample absorbance)/control absorbance] × 100. Dose-response curves (%DPPH scavenging vs. decadic logarithm concentration) were built to calculate the half-maximal inhibition concentration (IC_50_) through non-linear regression using GraphPad Prism 7.0 software (GraphPad, San Diego, CA, USA).

### 3.7. HPLC-ESI-MS Analysis

The analyses were performed using HPLC equipment (Shimadzu Prominence LC) (Shimadzu, Columbia, MD, USA) coupled with a TOF mass spectrometer (Bruker microTOFQ II) (Bruker, Billerica, MA, USA). A Phenomenex Luna-C18 column (250 × 4.6 × 5 µm) with mobile phases A (1% HCOOH in type-I water) and B (1% HCOOH in acetonitrile) in gradient elution an oven temperature of 40 °C was used. The gradient comprised 0–2 min 10% B, 10 min 15% B, 30 min 25% B, 34–36 min 80% B, 40–45 min 10% B. The spectra were recorded between 200 and 800 nm with a detection wavelength of 520 nm. The sample (5 µL) was injected through the autosampler. ESI interface of 1.3 kV in full scan positive mode in the range of 50–1000 amu, heating block at 400 °C, nebulizer, and drying gas (N_2_) 1.5 L/min and 8 L/min respectively, quadrupole energy of at 7.0 eV and collision energy of 14 eV. The compounds were annotated at level 3, based on confidence levels established for communicating metabolite identity through HRMS [62], by combining MS and HRMS data. This annotation process involved a comprehensive diagnostic analysis, considering factors such as accurate mass, quasimolecular ion, and MS fragments, and supported by phylogeny, chromatographic behavior, and comparison with available literature and KNApSAcK database (http://kanaya.naist.jp/knapsack_jsp/top.html, accessed on 28 December 2023). The anthocyanin quantification employed cyanidin-3-glucoside (C3G) (Sigma-Aldrich, St. Louis, MO, USA) as the external standard, monitored at 520 nm using a photodiode array (PDA) detector. Anthocyanin contents were expressed as mg C3G equivalents per 100 g of fresh blueberry (mg eq C3G/g fw). Corrected peak areas of detected anthocyanins were determined using relative response factors. Each analysis included three biological and two technical replicates to ensure accuracy. Method precision was evaluated via intra and inter-day analyses of C3G, yielding relative standard deviations (RSD %) of 2.8% and 3.7%, respectively. The Limit of Detection (LoD) and Limit of Quantification (LoQ) for LC-DAD analysis of C3G were determined as 320 ng/mL and 675 ng/mL, respectively, with recoveries ranging from 96.8% to 103.2%. Quality control was assured by injecting pooled samples to evaluate detector response consistency.

### 3.8. Statistical Analysis

Normality test (Shapiro-Wilks), analysis of variance (ANOVA), and Tukey’s test were applied to total content data, which were performed in InfoStat statistical software v29.09.2020 (National University of Córdoba, Córdoba, Argentina). The Tukey’s test for multiple comparisons employs pairwise post-hoc testing to ascertain whether there exists a significant difference between the means of all possible pairs, which are typically grouped by letters. This test is instrumental in identifying which means within a set differ significantly from one another. In addition, the whole analysis of quantitative data of individual anthocyanins was carried out by sparse partial-least-squares discriminant analysis (sPLS-DA) in the web tool MetaboAnalyst 5.0 [73] to identify different patterns between data sets.

## 4. Concluding Remarks

Research into the chemical characterization of blueberries is imperative, particularly for those cultivars introduced in certain environments such as the Andean plateau. This research aimed to distinguish three southern highbush blueberry cultivars and uncover valuable traits using biomarkers. The chemical profiles of locally cultivated and commercially available blueberries revealed particular differences in TPC, TFC, and TAC. However, the cultivated southern highbush blueberries exhibited similar patterns between them, with certain differences for the ‘Legacy’ cultivar. However, the targeted anthocyanin profiling-based differentiation of fruits from different types of blueberries exhibited the absence of three compounds in the dehydrated blueberry (DD). The supervised statistics through sPLS-DA analysis revealed a robust relationship between specific sets of anthocyanins found consistently across samples and those predominantly present two cultivars, i.e., cyanidin galactoside (**3**) was identified as a discriminant for the ‘Biloxi’ cultivar and petunidin glucoside (**6**) was relevant for the ‘Legacy’cultivar. This distinct set could potentially serve as a quality parameter for locally produced blueberry fruits since they can provide valuable insights into potential tools for authentication and quality assessments.

## Figures and Tables

**Table 1:** Annotated anthocyanins occurred in fruits of southern highbush blueberry cultivars.

# ^a^	t_R_^b^ (min)	[M]^+^ (*m/z*)	Aglycone (*m/z*)	Formula	Accurate Mass	Error ^c^ (ppm)	Annotation ^d^
**1**	12.01	465.1031	303.0530	C_21_H_21_O_12_	465.1034	0.432	delphinidin galactoside
**2**	12.88	465.1038	303.0526	C_21_H_21_O_12_	465.1034	1.073	delphinidin glucoside
**3**	14.25	449.1073	287.0584	C_21_H_21_O_11_	449.1084	2.419	cyanidin galactoside
**4**	15.24	449.1055	287.0576	C_28_H_17_O_6_	449.1026	6.427	cyanidin glucoside
**5**	15.54	479.1208	317.0691	C_22_H_23_O_12_	479.1190	3.859	petunidin galactoside
**6**	16.49	479.1178	317.0684	C_22_H_23_O_12_	479.1190	2.403	petunidin glucoside
**7**	17.87	449.1057	317.0685	C_28_H_17_O_6_	449.1026	5.982	petunidin arabinoside
**8**	18.96	493.1328	331.0845	C_23_H_25_O_12_	493.1347	3.653	malvidin galactoside
**9**	19.99	493.1358	331.0845	C_23_H_25_O_12_	493.1347	2.431	malvidin glucoside
**10**	21.50	463.121	331.0845	C_22_H_23_O_11_	463.1241	6.557	malvidin arabinoside

^a^ Compound numbering according to chromatogram (Figure 4); ^b^ t_R_ = retention time (min); ^c^ Relative error (in ppm) between HRMS-measured accurate mass and theoretical monoisotopic mass of the quasimolecular ion; ^d^ Annotated anthocyanins at level 3 according to the confidence levels to communicate metabolite identity by high-resolution mass spectrometry (HRMS) [62].

**Table 2:** Contents of each anthocyanin in fruits of southern highbush blueberries.

Type ^a^	Anthocyanin ^b^ (mg C3G/100 g fw) ^c^									
	1	2	3	4	5	6	7	8	9	10
SB	93.8 ± 1.5 ^C^	40.5 ± 0.7 ^B^	75.8 ± 1.1 ^D^	8.8 ± 0.3 ^D^	36.4 ± 0.4 ^D^	37.7 ± 0.8 ^D^	16.9 ± 0.6 ^D^	31.3 ± 0.4 ^E^	19.7 ± 0.6 ^C^	9.5 ± 0.5 ^D^
LG	79.8 ± 3.1 ^D^	55.3 ± 2.2 ^A^	73.0 ± 2.9 ^D^	4.3 ± 0.2 ^C^	46.4 ± 2.2 ^B^	55.1 ± 2.8 ^A^	29.9 ± 1.5 ^A^	73.1 ± 2.2 ^B^	56.5 ± 3.2 ^A^	42.1 ± 2.8 ^A^
BL	96.5 ± 0.7 ^B^	38.9 ± 0.6 ^C^	110.5 ± 1.3 ^A^	13.5 ± 0.3 ^A^	35.9 ± 0.2 ^D^	49.7 ± 0.1 ^B^	20.2 ± 0.5 ^C^	25.4 ± 1.0 ^F^	19.1 ± 0.8 ^C^	7.6 ± 0.1 ^E^
BF	80.6 ± 0.2 ^D^	28.6 ± 0.1 ^E^	101.2 ± 0.3 ^B^	11.0 ± 0.1 ^B^	41.0 ± 0.2 ^C^	49.4 ± 0.2 ^B^	30.3 ± 0.1 ^A^	61.5 ± 0.2 ^C^	35.9 ± 0.2 ^B^	34.3 ± 0.1 ^B^
OC	92.7 ± 3.7 ^BC^	34.7 ± 1.4 ^D^	59.7 ± 0.8 ^E^	4.3 ± 0.1 ^C^	54.7 ± 2.4 ^A^	44.1 ± 1.5 ^C^	22.8 ± 1.0 ^B^	88.2 ± 1.4 ^A^	56.5 ± 0.6 ^A^	35.3 ± 1.7 ^B^
DD	115 ± 6.8 ^A^	n.d.	90.3 ± 3.8 ^C^	n.d.	52.3 ± 3.4 ^A^	15.6 ± 0.5 ^E^	23.1 ± 0.9 ^B^	57.2 ± 2.1 ^D^	0.5 ± 0.1 ^D^	17.4 ± 1.0 ^C^

^a^ Blueberry fruits used for extract preparation and anthocyanin quantification. Commercial berries = Berry Fruit (BF), Delynat Dried (DD), and Ocati (OC); Locally cultivated southern highbush blueberry cultivars = ‘Legacy’ (LG), ‘Sharpblue’ (SB), and ‘Biloxi’ (BL). ^b^ Numbering of quantified anthocyanins according to the annotation in Table 1. ^c^ Contents expressed as mg eq cyanidin-3-glucoside (C3G) per 100 g fresh weight (fw); n.d. = not detected. Data expressed as means ± standard deviation (*n* = 6). Different superscript uppercase letters indicate significant differences per anthocyanin among fruit samples according to the Tukey test.

## Data Availability

The data presented in this study are available upon request from the corresponding author.
